# Beyond Linear Risk: A U-Shaped Association Between Platelet Reactivity and Mortality in Coronary Artery Disease

**DOI:** 10.3390/diseases14060194

**Published:** 2026-05-29

**Authors:** Sholpan Zhangelova, Orazbek Sakhov, Lyazat Abisheva, Dmitriy Polyakov, Farida Rustamova, Aizhan Almukhanova, Galiya Umenova, Gulzada Nurgaliyeva, Aigyul Izhanova, Dana Akhmentayeva, Dina Kapsultanova, Friba Nurmukhammad

**Affiliations:** 1 Faculty of Postgraduate Education, Asfendiyarov Kazakh National Medical University, Almaty 050012, Kazakhstan; zhangelova.s@kaznmu.kz (S.Z.); sakhov.o@kaznmu.kz (O.S.); rustamova.f@kaznmu.kz (F.R.); almukhanova.a@kaznmu.kz (A.A.); nurgaliyeva.g@kaznmu.kz (G.N.); izhanova.a@kaznmu.kz (A.I.); ahmentaeva.d@kaznmu.kz (D.A.); 2 Department of cardiology, City Cardiology Center, Almaty 050000, Kazakhstan; lyazat1@mail.ru (L.A.); umenova_g@mail.ru (G.U.); 3 Federal Stae Budgetary Educational Institution, Higher Education «Privolzhsky Research Medical University», Nizhny Novgorod 603005, Russia; polyakov_d@pimunn.net; 4 Faculty of Postgraduate Medical Education, Hodja Ahmed Yasawi International Kazakh-Turkish University, Turkestan 161200, Kazakhstan

**Keywords:** platelet reactivity, P2Y12 reaction units, cardiovascular mortality, non-linear association, restricted cubic spline, coronary artery disease

## Abstract

Background: Optimal platelet inhibition is essential for minimizing both thrombotic and hemorrhagic complications in patients with coronary artery disease (CAD). Although high on-treatment platelet reactivity (HPR) has been consistently associated with adverse clinical outcomes, the relationship between platelet reactivity—measured as P2Y12 reaction units (PRU)—and cardiovascular mortality remains incompletely characterized. In particular, potential non-linear associations have not been adequately explored. Objective: We aimed to investigate the association between PRU and cardiovascular mortality in patients with CAD, with a specific focus on identifying potential non-linear relationships. Methods: We conducted a retrospective observational cohort study including 1000 patients with angiographically confirmed CAD treated at a tertiary cardiology center in Almaty, Kazakhstan, between 2024 and 2025. Platelet reactivity was assessed using the VerifyNow P2Y12 assay. Multivariable logistic regression models were used to identify independent predictors of cardiovascular mortality. To assess potential non-linear associations between PRU and mortality, restricted cubic spline regression was applied with predefined knot placement. Model performance was evaluated in terms of discrimination (C-statistic) and calibration (Hosmer-Lemeshow goodness-of-fit test). Results: In conventional linear regression models, PRU was not independently associated with cardiovascular mortality (odds ratio [OR] ~1.00; *p* > 0.05). However, spline-based analyses demonstrated a statistically significant non-linear (U-shaped) relationship between PRU and mortality risk (*p* for non-linearity = X). Both low and high PRU values were associated with increased mortality, whereas intermediate PRU levels corresponded to the lowest observed risk. Additional independent predictors of mortality included advanced age, diabetes mellitus, and elevated inflammatory markers. Conclusions: Our findings reveal a significant non-linear association between platelet reactivity and cardiovascular mortality in patients with CAD. Both insufficient and excessive platelet inhibition appear to confer increased risk, suggesting that optimal PRU targets may lie within an intermediate therapeutic range. These results support a paradigm shift toward more individualized antiplatelet therapy strategies guided by platelet function testing.

## 1. Introduction

Platelets are central mediators of arterial thrombosis, and their excessive activation following atherosclerotic plaque rupture drives acute coronary syndrome (ACS) and ischemic complications after percutaneous coronary intervention (PCI) [1]. Conversely, overly aggressive platelet inhibition increases bleeding risk, a complication with its own mortality burden. Accordingly, characterizing the full spectrum of platelet reactivity and its relationship with clinical outcomes is of paramount therapeutic importance [2,3]. Residual high on-treatment platelet reactivity (HRPR), defined as PRU ≥ 208 by the VerifyNow assay, has been associated with a two- to ninefold increase in stent thrombosis risk and recurrent ischemic events [4,5,6]. Multiple patient-level factors contribute to HRPR, including advanced age, diabetes mellitus, chronic kidney disease (CKD), obesity, and CYP2C19 loss-of-function polymorphisms [7,8,9]. Importantly, patients over 70 years of age are two to three times more likely to exhibit HRPR compared with younger counterparts [10,11]. Despite this established risk, the prognostic utility of PRU as a continuous variable remains controversial [12]. Prior studies have predominantly examined high platelet reactivity thresholds, with limited attention to the potential harms of excessive platelet inhibition. Emerging evidence suggests that very low PRU values may also confer risk, implying the existence of an optimal therapeutic window, yet this has not been systematically evaluated across antiplatelet therapy strategies in a real-world cohort. The primary aim of this study was to identify independent predictors of all-cause mortality in patients with CAD receiving antiplatelet therapy and, secondarily, to characterize the shape of the association between PRU and mortality across the full range of platelet reactivity values [13,14].

Importantly, while high on-treatment platelet reactivity (HRPR) is established as a risk factor for ischemic events, excessively low PRU values—reflecting over-inhibition of platelet function—have been independently associated with increased bleeding risk, including gastrointestinal and intracranial hemorrhage, which carries its own mortality burden [15]. The existence of this dual risk implies that the optimal clinical target may lie within an intermediate therapeutic window rather than at maximal suppression. Despite this, the full PRU continuum—including very low values—has rarely been simultaneously evaluated in a single real-world cohort [15,16].

Objective: We aimed to evaluate the association between PRU and cardiovascular mortality in patients with CAD and to explore potential non-linear relationships.

## 2. Materials and Methods

### 2.1. Study Design and Setting

**Figure 1:**
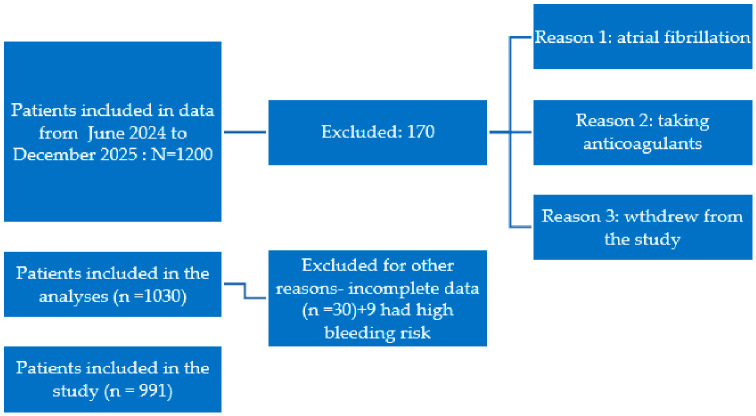
Flow chart of patients.

A retrospective cohort study was conducted using medical records of 1000 adult patients (≥18 years) with established coronary artery disease who received care at a cardiology center in Almaty, Kazakhstan, between June 2024 and December 2025. Patients were enrolled if they had ischemic heart disease and were receiving single or dual antiplatelet therapy. The exclusion criteria included atrial fibrillation with anticoagulant use (rivaroxaban, apixaban, or dabigatran), coagulopathy, and high bleeding risk. Nine patients with high bleeding risk (PRU < 95) were excluded from the primary analysis per a pre-specified study protocol, based on their clinical designation as high-bleeding-risk according to consensus VerifyNow P2Y12 thresholds (Figure 1).

As a sensitivity analysis, all primary analyses were repeated in the full cohort of 1000 patients, including the 9 patients with PRU < 95. The results were consistent with the primary analysis and are presented in Table A1. The exclusion of these patients from the primary analysis was a pre-specified protocol decision based on their clinical designation as high-bleeding-risk per institutional guidelines, not a post hoc statistical decision.

The primary outcome was cardiovascular mortality, defined as death attributable to a cardiovascular cause occurring during the observation period. Cause of death was ascertained from electronic hospital records, discharge summaries, and, where applicable, death certificates obtained from the regional civil registry. All deaths were reviewed and classified by two independent cardiologists who were blinded to the patients’ PRU values. Cardiovascular deaths included fatal acute myocardial infarction, sudden cardiac death, fatal heart failure, and fatal stroke. Deaths attributable to non-cardiovascular causes (infection, malignancy, or trauma) were excluded from the primary outcome. Deaths from major bleeding events were classified as hemorrhagic deaths and not counted under the primary cardiovascular mortality endpoint. No independent adjudication committee was established, which we acknowledge as a limitation. However, dual independent physician review was performed for all mortality events, and inter-rater disagreements were resolved by consensus.

### 2.2. Platelet Reactivity Assessment

Residual platelet reactivity (RPR) was evaluated using the VerifyNow P2Y12 assay (Instrumentation Laboratory, Bedford, MA, USA), a point-of-care system validated for quantifying P2Y12 receptor-mediated platelet inhibition. PRU thresholds were applied according to international consensus guidelines: HRPR was defined as PRU ≥ 208, therapeutic window as PRU 95-208, and high bleeding risk as PRU < 95.

### 2.3. Clinical and Laboratory Data Collection

Baseline demographic, clinical, and pharmacological data were extracted from electronic medical records. The variables systematically evaluated included lipid profile (total cholesterol, LDL-C, HDL-C, and triglycerides); renal function (serum creatinine and eGFR); hemoglobin (with anemia defined by WHO criteria); left ventricular ejection fraction (LVEF) by transthoracic echocardiography; body mass index (BMI); inflammatory and coagulation markers (CRP, fibrinogen, D-dimer, and troponin I); and cardiovascular comorbidities (heart failure, diabetes mellitus, prior myocardial infarction, and obstructive CAD).

### 2.4. Ethical Consideration

This study was conducted in accordance with the ethical principles outlined in the Declaration of Helsinki. Written informed consent was obtained from all participants prior to inclusion. The study protocol was reviewed and approved by the institutional ethics committee of Hodja Ahmed Yasawi International Kazakh-Turkish University (Protocol No. 53; dated 17 June 2024).

### 2.5. Statistical Analysis

Continuous variables are presented as medians and interquartile ranges [IQRs], as normality assumptions were not met based on the Shapiro-Wilk test. Between-group comparisons were performed using the Mann-Whitney U test for continuous variables and the chi-square test or Fisher’s exact test, as appropriate, for categorical variables. To identify independent predictors of cardiovascular mortality, multivariable logistic regression analysis was performed. Variables with a *p*-value of <0.10 in univariable analyses were considered for inclusion in the multivariable model. The results are reported as odds ratios (ORs) with corresponding 95% confidence intervals (CIs). To explore potential non-linear associations between platelet reactivity and mortality, PRU values were initially categorized into quartiles. Additionally, restricted cubic spline regression models were constructed with 3–5 knots placed at prespecified percentiles to flexibly model the relationship between PRU and mortality risk. Non-linearity was formally tested using likelihood ratio tests. The probability density distributions of PRU were examined across quartiles within subgroups defined by antiplatelet therapy to further characterize distributional patterns. Model performance was evaluated in terms of discrimination and calibration using the C-statistic and the Hosmer-Lemeshow goodness-of-fit test, respectively. All statistical tests were two-sided, and a *p*-value < 0.05 was considered statistically significant. Statistical analyses were performed using R software (version 4.x).

The use of logistic regression was dictated by the structure of the available data; individual time-to-event variables—specifically, the exact date of death for each patient—were not available in the source database. Cox regression requires a precise time-to-event variable for each observation, including censored cases (patients alive at the study end). Since this information was not captured during retrospective data extraction from electronic medical records, multivariable logistic regression was the only feasible method of multivariable analysis for a binary outcome, given the available data structure. Future prospective studies with systematic recording of exact event dates should employ Cox proportional hazards regression and Cox spline models. For the analysis of nonlinearity, logistic regression with restricted cubic splines was applied, consistent with the binary nature of the outcome; the results are qualitatively consistent with supplementary analyses performed using quartile and rank-based approaches.

## 3. Results

A total of 1000 patients were included in the analysis; 856 (85.6%) survived, and 144 (14.4%) died during the observation period due to cardiovascular events. Non-survivors were significantly older than survivors (median: 74.0 [67.0–79.0] vs. 65.0 [59.0–72.0] years; OR: 1.09 (95% CI: 1.07–1.12); *p* < 0.001) and were more frequently female (58.3% vs. 43.6%; OR: 0.55 for males (95% CI: 0.38–0.79); *p* = 0.001). Non-survivors demonstrated significantly impaired renal function (eGFR: 68.0 [55.0–84.0] vs. 77.0 [65.0–91.0] mL/min/1.73 m^2^; *p* < 0.001), lower hemoglobin (134 [123–146] vs. 141 [130–151] g/L; *p* < 0.001), and reduced LVEF (56.0 [45.0–62.2] vs. 59.0 [51.0–64.0]%; *p* = 0.004). Inflammatory markers—CRP (4.40 vs. 3.50 mg/L; *p* = 0.030) and fibrinogen (3.38 vs. 3.17 g/L; *p* = 0.021)—were elevated in non-survivors. Obstructive CAD was more prevalent in non-survivors (62.5% vs. 51.3%; OR: 1.58 (95% CI: 1.10–2.29); *p* = 0.013), as was a history of prior myocardial infarction (37.5% vs. 29.1%; OR: 1.46 (95% CI: 1.01–2.11); *p* = 0.046). A statistically significant but modest difference in PRU was observed between groups (120 [111–190] vs. 155 [120–200]; *p* = 0.046) (Table 1 and Table A2).

### 3.1. Multivariable Logistic Regression Analysis

Multivariable logistic regression was performed in 986 patients (Tjur R^2^ = 0.141). Advanced age remained the strongest independent predictor of mortality, with each additional year associated with an 8% increase in the odds of death (OR: 1.08 (95% CI: 1.06–1.11); *p* < 0.001). Male sex was independently associated with a lower risk of mortality (OR: 0.53 (95% CI: 0.33–0.86); *p* = 0.011). Reduced LVEF was an independent predictor of adverse outcome (OR: 0.98 per unit increase (95% CI: 0.96–1.00); *p* = 0.016). PRU reached statistical significance in the multivariable model (OR: 1.00 (95% CI: 0.99–1.00); *p* = 0.033), indicating that its effect is not captured by a simple linear term, consistent with the non-linear relationship described below. Obstructive CAD demonstrated borderline significance (OR: 1.46 (95% CI: 0.93–2.31); *p* = 0.098) (Table 2).

### 3.2. PRU Subgroup Analysis: Therapeutic Window vs. HRPR

After excluding nine patients with high bleeding risk, 991 patients were analyzed: 883 (89.1%) within the therapeutic window and 108 (10.9%) with HRPR. Baseline characteristics—including age, sex, renal function, BMI, inflammatory markers, and LVEF—were comparable between groups (all *p* > 0.05). Critically, mortality rates did not differ significantly between patients in the therapeutic window and those with HRPR (14.7% vs. 13.0%; OR: 0.87 (95% CI: 0.46–1.53); *p* = 0.643). HRPR was strongly associated with less-intensive antiplatelet therapy: ticagrelor use was markedly lower in the HRPR group (5.6% vs. 26.0%; OR: 0.17 (95% CI: 0.07–0.36); *p* < 0.001), and DAPT was less frequent (46.3% vs. 61.2%; OR: 0.55 (95% CI: 0.37–0.82); *p* = 0.003), reflecting expected pharmacodynamic differences (Table 3 and Table A3).

### 3.3. U-Shaped Relationship Between PRU Quartiles and Mortality: A Key Finding

A consistent non-linear (U-shaped) relationship between PRU and mortality was identified across all antiplatelet therapy subgroups, the primary novel finding of this study. In the monotherapy subgroup (*n* = 402), patients were stratified into quartiles (Q1: PRU ~120, Q2: PRU ~155, Q3: PRU ~190, and Q4: PRU ~220). The highest mortality was observed in Q1 (16.0%), with progressively lower rates in Q2 (7.9%) and Q3 (14.3%), and partial recovery in Q4 (11.0%). Although overall quartile differences did not reach statistical significance (*p* = 0.491), the bimodal pattern—with mortality peaks at both extremes of the PRU distribution—was numerically pronounced and clinically meaningful (Table A4).

In the DAPT subgroup (*n* = 598), this pattern was even more pronounced: Q1 mortality was 19.2%, compared with 11.7% (Q2), 12.8% (Q3), and 11.2% (Q4). Notably, this gradient approached statistical significance (*p* = 0.136). Importantly, the pattern was consistent regardless of whether patients received clopidogrel or ticagrelor, arguing against a drug-specific effect (Table 4).

**Figure 2:**
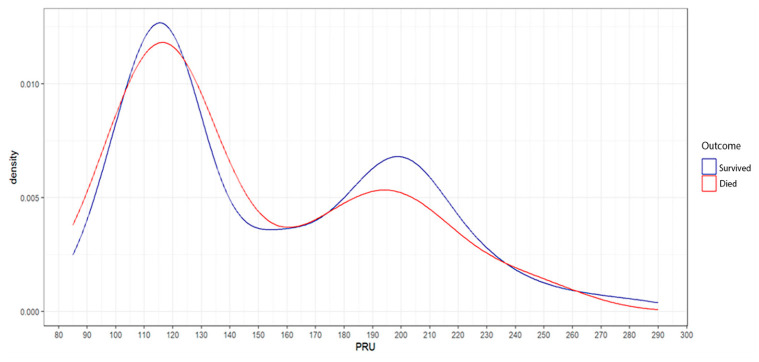
Probability density of mortality outcome in monotherapy subgroup.

**Figure 3:**
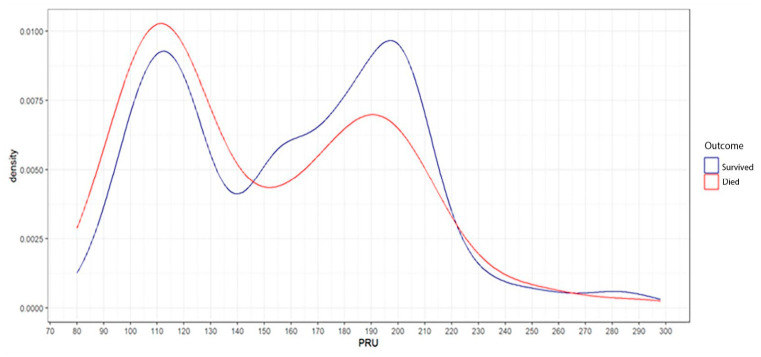
Probability density distribution of P2Y12 reaction units (PRU) in patients who died (non-survivors) and patients who survived (survivors) in the dual antiplatelet therapy subgroup.

Probability density analyses confirmed a bimodal distribution of mortality events across PRU values, with a dominant peak in the lowest PRU range. This pattern persisted across all treatment subgroups, supporting the hypothesis that very low platelet reactivity—representing excessive platelet inhibition—is independently associated with adverse outcomes, irrespective of the antiplatelet agent used (Figure 2 and Figure 3).

Probability density distribution of P2Y12 reaction units (PRU) in patients who died (non-survivors) and patients who survived (survivors) in the dual antiplatelet therapy (DAPT) subgroup. The *x*-axis represents PRU values; the *y*-axis represents the probability density. Separate curves are shown for survivors (blue) and non-survivors (red). No confidence interval shaded area is displayed; curves represent kernel density estimates (Figure 3).

Figure 2: Probability density distribution of P2Y12 reaction units (PRU) in patients who died (non-survivors) and patients who survived (survivors) in the monotherapy subgroup. The *x*-axis represents PRU values; the *y*-axis represents the probability density. Separate curves are shown for survivors (blue) and non-survivors (red). No confidence interval shaded area is displayed; curves represent kernel density estimates. Probability density distribution of P2Y12 reaction units (PRU) in patients who died (non-survivors) and patients who survived (survivors) within the monotherapy subgroup. The *x*-axis represents PRU values, and the *y*-axis represents the probability density. Separate curves are shown for survivors (blue) and non-survivors (red). No confidence interval shading is displayed; the curves represent kernel density estimates.

### 3.4. PRU-Stratified Analysis: PRU < 155 vs. PRU ≥ 155

To further characterize mortality risk at low and higher PRU values, patients were dichotomized at PRU 155 (cohort median). In the low-PRU subgroup (*n* = 454), non-survivors were significantly older (70.5 vs. 66.0 years; OR: 1.06 (95% CI: 1.04–1.09); *p* < 0.001), had a lower eGFR (*p* = 0.004), reduced hemoglobin (*p* = 0.034), and elevated inflammatory markers (CRP *p* = 0.036; fibrinogen *p* = 0.048). PRU itself did not differ between survivors and non-survivors within this stratum (*p* = 0.700), indicating that conventional risk factors—rather than PRU per se—drive mortality in this group. In the high-PRU subgroup (*n* = 546), age, female sex, impaired renal function, low LVEF, low triglycerides, and prior myocardial infarction were significant predictors of mortality, while PRU again did not differ between outcome groups (*p* = 0.902) (Table A1 and Table A5). This paradoxical finding reinforces the concept that the observed U-shaped relationship is a population-level phenomenon reflecting extreme platelet reactivity at both poles, rather than a simple dose-response effect of PRU within strata. Importantly, the within-stratum absence of PRU differences between survivors and non-survivors does not refute the population-level U-shaped association; rather, it is the expected result of analyzing a non-linear relationship within pre-defined subgroups restricted to narrow portions of the distribution. The U-shaped pattern is a phenomenon of the full PRU continuum, and it persists when mortality rates are normalized against the density of the overall PRU distribution (Figure 3).

### 3.5. Antiplatelet Therapy and Mortality

All cases of mortality were due to cardiovascular events. No significant association was observed between antiplatelet therapy type and mortality. Death rates were comparable between patients receiving clopidogrel and those not receiving it (15.5% vs. 13.8%) and between ticagrelor-treated and non-ticagrelor patients (13.6% vs. 14.7%). Monotherapy and DAPT were similarly associated with mortality (13.9% vs. 14.7%; *p* = 0.799). These findings indicate that the PRU-mortality relationship is independent of antiplatelet drug choice.

## 4. Discussion

In this large real-world cohort of 1000 patients with coronary artery disease (CAD), we demonstrate that the association between platelet reactivity and cardiovascular mortality is distinctly non-linear, following a U-shaped pattern. Mortality risk was increased at both extremes of the PRU distribution, with the highest risk consistently observed in the lowest PRU quartile across all treatment subgroups, including monotherapy, dual antiplatelet therapy (DAPT), and both clopidogrel- and ticagrelor-based regimens [15,16,17]. These findings suggest that excessive platelet inhibition may be as clinically detrimental as insufficient inhibition. Notably, conventional linear models failed to detect this relationship, whereas spline-based analyses revealed the underlying risk structure, underscoring the importance of modeling non-linearity in biomarker-outcome associations [18,19].

These observations have important mechanistic and clinical implications. Current antiplatelet strategies are largely oriented toward reducing thrombotic risk through intensified platelet inhibition. However, the potential adverse consequences of excessive platelet suppression, including bleeding complications, impaired vascular repair, and broader pleiotropic effects, have received comparatively less emphasis [20]. Our findings provide real-world evidence that very low PRU values are associated with a meaningful increase in mortality risk, challenging the prevailing assumption that lower platelet reactivity is uniformly beneficial [13].

The concept of an optimal therapeutic window of platelet reactivity is biologically plausible and supported by prior evidence. High on-treatment platelet reactivity (HPR) has been consistently associated with ischemic events, whereas low platelet reactivity has been linked to increased bleeding risk, including gastrointestinal and intracranial hemorrhage [2,3,4]. Our study extends this paradigm by demonstrating that the mortality impact of extreme PRU values is consistent across different antiplatelet agents, suggesting that the observed U-shaped relationship reflects a fundamental biological phenomenon rather than a drug-specific effect.

Several independent predictors of mortality identified in our cohort—advanced age, female sex [1], reduced left ventricular ejection fraction (LVEF) [12], obstructive CAD [6], and renal dysfunction—are consistent with the established literature [5,21]. Importantly, the non-linear association between PRU and mortality persisted after adjustment for these factors, indicating that platelet reactivity provides incremental prognostic information beyond traditional risk markers. The observed inverse association between smoking and mortality in the univariable analysis likely reflects residual confounding or selection bias (commonly referred to as the “smoker’s paradox”) rather than a true protective effect [7].

This study has several strengths, including a relatively large sample size, inclusion of diverse antiplatelet therapy strategies, and the consistent reproducibility of the U-shaped association across subgroups. The use of the VerifyNow P2Y12 assay, a widely validated and guideline-endorsed tool, enhances the clinical applicability of our findings. From a translational perspective, these results support a shift toward individualized antiplatelet therapy. Rather than adopting a uniform strategy of treatment intensification or de-escalation, clinicians may need to target an optimal range of platelet reactivity, particularly in patients with competing ischemic and bleeding risks.

## 5. Limitations

Several limitations should be acknowledged. First, the observational and single-center design limits causal inference. Second, platelet reactivity was assessed at a single time point, which may not capture temporal variability related to treatment changes or disease progression. Third, the use of logistic regression rather than Cox survival analysis represents a methodological limitation. Although follow-up was complete within the study window, future studies with prospective time-to-event data collection should employ proportional hazards regression and spline-based Cox models. Despite multivariable adjustment, residual confounding due to unmeasured variables cannot be excluded. Fourth, the study endpoint was confined to cardiovascular mortality; non-fatal cardiovascular events were not included, which may limit the interpretation of the observed U-shaped association between platelet reactivity and outcomes. The absence of a formal independent outcome adjudication committee represents a limitation, and the possibility of residual misclassification of cause of death cannot be entirely excluded. Additionally, because deaths from bleeding were not classified as cardiovascular events, this study may underestimate the full mortality burden attributable to low PRU in patients with excessive platelet inhibition. Finally, the study population represents a single Central Asian center, and external generalizability to other populations and ethnic groups requires further validation.

## 6. Conclusions

In a real-world cohort of 1000 patients with coronary artery disease receiving antiplatelet therapy, platelet reactivity demonstrated a robust non-linear (U-shaped) association with cardiovascular mortality, with increased risk at both the lowest and highest ends of the PRU spectrum, irrespective of the antiplatelet regimen used. Conventional linear regression did not capture this relationship, highlighting the importance of spline-based modeling in biomarker-outcome analyses. Advanced age, female sex, reduced LVEF, and obstructive CAD were independently associated with mortality. These findings support the concept of an optimal therapeutic window of platelet reactivity and challenge the paradigm of maximal uniform platelet suppression. Prospective studies are warranted to define target PRU ranges and evaluate whether platelet function-guided antiplatelet therapy improves clinical outcomes in patients with coronary artery disease.

## Figures and Tables

**Table 1:** Baseline characteristics and univariable predictors of mortality.

Variable	Survivors (*n* = 856)	Non-Survivors (*n* = 144)	OR (95% CI)	*p*-Value
Age, years	65.0 [59.0–72.0]	74.0 [67.0–79.0]	1.09 [1.07–1.12]	<0.001
Male sex	483 (56.4%)	60 (41.7%)	0.55 [0.38–0.79]	0.001
eGFR, mL/min/1.73 m^2^	77.0 [65.0–91.0]	68.0 [55.0–84.0]	0.98 [0.97–0.99]	<0.001
Hemoglobin, g/L	141 [130–151]	134 [123–146]	0.98 [0.97–0.99]	<0.001
LVEF, %	59.0 [51.0–64.0]	56.0 [45.0–62.2]	0.98 [0.96–0.99]	0.004
CRP, mg/L	3.50 [2.20–6.14]	4.40 [2.66–8.40]	1.01 [1.00–1.01]	0.03
Fibrinogen, g/L	3.17 [2.80–3.90]	3.38 [3.00–4.04]	1.23 [1.03–1.46]	0.021
Triglycerides, mmol/L	1.30 [1.10–1.80]	1.29 [0.99–1.57]	0.66 [0.52–0.85]	0.001
Obstructive CAD	439 (51.3%)	90 (62.5%)	1.58 [1.10–2.29]	0.013
Prior MI	249 (29.1%)	54 (37.5%)	1.46 [1.01–2.11]	0.046
Smoking	156 (18.2%)	15 (10.4%)	0.53 [0.29–0.90]	0.017
PRU	155 [120–200]	120 [111–190]	1.00 [0.99–1.00]	0.046

CAD = coronary artery disease; CRP = C-reactive protein; eGFR = estimated glomerular filtration rate; LVEF = left ventricular ejection fraction; MI = myocardial infarction; PRU = P2Y12 reaction units.

**Table 2:** Multivariable logistic regression model for mortality.

Variable	OR (95% CI)	*p*-Value
Age (per year)	1.08 [1.06–1.11]	<0.001
Male sex	0.53 [0.33–0.86]	0.011
LVEF (per unit)	0.98 [0.96–1.00]	0.016
PRU (per unit)	1.00 [0.99–1.00]	0.033
Obstructive CAD	1.46 [0.93–2.31]	0.098
Total cholesterol	0.77 [0.58–1.02]	0.076
Observations	986	
Tjur R^2^	0.141	

Only variables with *p* < 0.15 are shown. LVEF = left ventricular ejection fraction; PRU = P2Y12 reaction units.

**Table 3:** Comparison of patients within the therapeutic window vs. high on-treatment platelet reactivity (HRPR).

Variable	Therapeutic Window (*n* = 883)	HRPR (*n* = 108)	OR (95% CI)	*p*-Value
Age, years	67.0 [60.0–73.0]	66.5 [59.0–75.0]	1.00 [0.98–1.02]	0.749
Male sex	482 (54.6%)	57 (52.8%)	0.93 [0.62–1.39]	0.722
Mortality	130 (14.7%)	14 (13.0%)	0.87 [0.46–1.53]	0.643
PRU	150 [120–188]	230 [217–255]	-	<0.001
DAPT use	540 (61.2%)	50 (46.3%)	0.55 [0.37–0.82]	0.003
Ticagrelor use	230 (26.0%)	6 (5.6%)	0.17 [0.07–0.36]	<0.001

DAPT = dual antiplatelet therapy; HRPR = high on-treatment platelet reactivity; PRU = P2Y12 reaction units.

**Table 4:** Mortality rates across PRU quartiles by antiplatelet therapy subgroup.

Subgroup	Q1 (Lowest PRU)	Q2	Q3	Q4 (Highest PRU)
Monotherapy (*n* = 402)	16.00%	7.90%	14.30%	11.00%
DAPT (*n* = 598)	19.20%	11.70%	12.80%	11.20%
Median PRU (approx.)	~120	~155	~190	~220

Mortality rates are shown as a percentage of patients in each quartile. Q1 = lowest platelet reactivity; Q4 = highest platelet reactivity. DAPT = dual antiplatelet therapy.

## Data Availability

The raw data supporting the conclusions of this article will be made available by the authors upon request.

## Abbreviations

The following abbreviations are used in this manuscript:

ACS	Acute coronary syndrome
BMI	Body mass index
CAD	Coronary artery disease
CRP	C-reactive protein
GFR	Glomerular filtration rate
HRPR	High residual platelet reactivity
LDL-C	Low-density lipoprotein cholesterol
HDL-C	High-density lipoprotein cholesterol
MACEs	Major adverse cardiovascular events
RPR	Residual platelet reactivity
TGs	Triglycerides
PRU	P2Y12 reaction units
DAPT	Dual antiplatelet therapy
CKD	Chronic kidney disease
MI	Myocardial infarction
STEMI	ST-elevation myocardial infarction
WHO	World Health Organization
LVEF	Left ventricular ejection fraction
COPD	Chronic obstructive pulmonary disease
HTPR	High on-treatment platelet reactivity
HFpEF	Heart failure with preserved ejection fraction
ASA	Acetylsalicylic acid

## Appendix A

**Table A1:** Differences between survivors and non-survivors in the PRU < 155 subgroup.

Variable	Survived (*N* = 376)	Died (*N* = 78)	OR	*p* (Univariate)	*p* (Overall)	*N*
Age	66.0 [58.8–72.0]	70.5 [65.2–78.0]	1.06 [1.04–1.09]	<0.001	<0.001	454
Sex				0.054		454
Female	169 (44.9%)	45 (57.7%)	Reference	Reference		
Male	207 (55.1%)	33 (42.3%)	0.60 [0.36–0.98]	0.042		
eGFR	77.0 [64.0–90.5]	71.0 [59.0–83.8]	0.98 [0.97–0.99]	0.004	0.006	453
BMI	28.0 [25.0–31.3]	27.9 [25.0–31.0]	0.97 [0.93–1.02]	0.268	0.349	454
CRP	3.33 [2.20–5.72]	4.35 [3.11–6.80]	1.01 [1.00–1.03]	0.139	0.036	451
Total cholesterol	4.90 [3.99–5.73]	4.81 [4.08–5.78]	0.95 [0.78–1.15]	0.585	0.641	453
LDL-C	3.01 [2.20–3.74]	3.04 [2.16–3.83]	1.00 [0.81–1.24]	0.990	0.749	454
Triglycerides	1.30 [1.09–1.61]	1.30 [1.11–1.82]	0.91 [0.70–1.18]	0.479	0.436	454
Fibrinogen	3.17 [2.84–3.93]	3.38 [3.00–4.03]	1.19 [0.92–1.54]	0.175	0.048	454
Type 2 diabetes				0.104		454
No	291 (77.4%)	53 (67.9%)	Reference	Reference		
Yes	85 (22.6%)	25 (32.1%)	1.62 [0.94–2.74]	0.084		
Prediabetes				0.481		454
No	363 (96.5%)	77 (98.7%)	Reference	Reference		
Yes	13 (3.46%)	1 (1.28%)	0.41 [0.02–2.12]	0.343		
COVID-19 history				0.930		454
No	304 (80.9%)	64 (82.1%)	Reference	Reference		
Yes	72 (19.1%)	14 (17.9%)	0.93 [0.48–1.71]	0.824		
COPD				0.475		454
No	366 (97.3%)	75 (96.2%)	Reference	Reference		
Yes	10 (2.66%)	3 (3.85%)	1.51 [0.32–5.18]	0.561		
Coronary artery disease severity				0.109		454
Non-obstructive	185 (49.2%)	30 (38.5%)	Reference	Reference		
Obstructive	191 (50.8%)	48 (61.5%)	1.55 [0.94–2.57]	0.085		
D-dimer	200 [121–480]	220 [148–588]	1.00 [1.00–1.00]	0.029	0.252	453
Troponin	0.002 [0.001–0.006]	0.003 [0.001–0.008]	0.98 [0.93–1.04]	0.556	0.090	454
LVEF (%)	59.0 [51.0–63.2]	57.0 [52.0–64.8]	0.99 [0.97–1.01]	0.529	0.692	454
Smoking				0.902		454
No	318 (84.6%)	67 (85.9%)	Reference	Reference		
Yes	58 (15.4%)	11 (14.1%)	0.91 [0.43–1.77]	0.789		
Hemoglobin	142 [131–152]	136 [124–154]	0.99 [0.97–1.00]	0.034	0.043	454
Prior MI				0.572		454
No	261 (69.4%)	51 (65.4%)	Reference	Reference		
Yes	115 (30.6%)	27 (34.6%)	1.20 [0.71–2.01]	0.485		
PRU	120 [105–120]	120 [101–120]	1.00 [0.97–1.02]	0.700	0.735	454
Statin therapy			[121–480]	0.200		454
Statin	349 (92.8%)	76 (97.4%)	Reference	Reference		
Statin + ezetimibe	27 (7.18%)	2 (2.56%)	0.36 [0.05–1.26]	0.122		
HRPR				0.369		454
Bleeding risk	9 (2.39%)	0 (0.00%)	Reference	Reference		
Therapeutic window	367 (97.6%)	78 (100%)	-	-		
Antiplatelet therapy:				0.440		454
Monotherapy	180 (47.9%)	33 (42.3%)	Reference	Reference		
Dual therapy	196 (52.1%)	45 (57.7%)	1.25 [0.76–2.06]	0.374		
Ticagrelor use				0.507		454
No	277 (73.7%)	54 (69.2%)	Reference	Reference		
Yes	99 (26.3%)	24 (30.8%)	1.25 [0.72–2.11]	0.424		
Clopidogrel use				0.949		454
No	279 (74.2%)	57 (73.1%)	Reference	Reference		
Yes	97 (25.8%)	21 (26.9%)	1.06 [0.60–1.83]	0.827		

Abbrevations: GFR = glomerular filtration rate, CRP = C-reactive protein, BMI = body mass index, LDL-C = low-density lipoprotein cholesterol, COPD = Chronic obstructive pulmonary disease, LVEF = left ventricular ejection fraction, MI = myocardial infarction, PRU = P2Y12 reaction units, HRPR = high residual platelet reactivity.

**Table A2:** Full baseline characteristics: survivors vs. non-survivors (*n* = 1000).

Variable	Survivors (*n* = 856)	Non-Survivors (*n* = 144)	OR (95% CI)	*p*-Value
Age, years	65.0 [59.0–72.0]	74.0 [67.0–79.0]	1.09 [1.07–1.12]	<0.001
Male sex	483 (56.4%)	60 (41.7%)	0.55 [0.38–0.79]	0.001
eGFR, mL/min/1.73 m^2^	77.0 [65.0–91.0]	68.0 [55.0–84.0]	0.98 [0.97–0.99]	<0.001
BMI, kg/m^2^	28.0 [25.0–31.2]	27.3 [25.0–30.0]	0.96 [0.92–0.99]	0.014
Hemoglobin, g/L	141 [130–151]	134 [123–146]	0.98 [0.97–0.99]	<0.001
LVEF, %	59.0 [51.0–64.0]	56.0 [45.0–62.2]	0.98 [0.96–0.99]	0.008
CRP, mg/L	3.50 [2.20–6.14]	4.40 [2.66–8.40]	1.01 [1.00–1.01]	0.009
Fibrinogen, g/L	3.17 [2.80–3.90]	3.38 [3.00–4.04]	1.23 [1.03–1.46]	0.003
D-dimer	220 [130–500]	226 [150–665]	1.00 [1.00–1.00]	0.163
Troponin I	0.00 [0.00–0.01]	220 [130–500]	0.99 [0.98–1.01]	0.021
Total cholesterol, mmol/L	4.90 [4.02–5.78]	4.74 [3.86–5.60]	0.87 [0.75–1.00]	0.069
LDL-C, mmol/L	3.08 [2.33–3.84]	2.96 [2.20–3.77]	0.91 [0.77–1.07]	0.151
Triglycerides, mmol/L	1.30 [1.10–1.80]	1.29 [0.99–1.57]	0.66 [0.52–0.85]	0.001
Type 2 diabetes	208 (24.3%)	43 (29.9%)	1.33 [0.89–1.95]	0.187
Prediabetes	28 (3.3%)	3 (2.1%)	0.66 [0.15–1.90]	0.606
COVID-19 history	177 (20.7%)	27 (18.8%)	0.89 [0.56–1.38]	0.675
COPD	30 (3.5%)	6 (4.2%)	1.22 [0.45–2.81]	0.879
Obstructive CAD	439 (51.3%)	90 (62.5%)	1.58 [1.10–2.29]	0.016
Prior MI	249 (29.1%)	54 (37.5%)	1.46 [1.01–2.11]	0.053
Smoking	156 (18.2%)	15 (10.4%)	0.53 [0.29–0.90]	0.029
PRU	155 [120–200]	120 [111–190]	1.00 [0.99–1.00]	0.038
HRPR (PRU ≥ 208)	94 (11.0%)	14 (9.7%)	-	0.601
Monotherapy	346 (40.4%)	56 (38.9%)	Reference	0.799
DAPT	510 (59.6%)	88 (61.1%)	1.07 [0.74–1.54]	0.733
Ticagrelor	210 (24.5%)	33 (22.9%)	0.92 [0.60–1.38]	0.754
Clopidogrel	300 (35.1%)	55 (38.2%)	1.14 [0.79–1.64]	0.531
Statin + ezetimibe	63 (7.4%)	7 (4.9%)	0.66 [0.27–1.37]	0.362

BMI = body mass index; CAD = coronary artery disease; COPD = chronic obstructive pulmonary disease; CRP = C-reactive protein; DAPT = dual antiplatelet therapy; eGFR = estimated glomerular filtration rate; HRPR = high on-treatment platelet reactivity; LDL-C = low-density lipoprotein cholesterol; LVEF = left ventricular ejection fraction; MI = myocardial infarction; PRU = P2Y12 reaction units.

**Table A3:** Full comparison: therapeutic window vs. HRPR (*n* = 991).

Variable	Therapeutic Window (*n* = 883)	HRPR (*n* = 108)	OR (95% CI)	*p*-Value
Age, years	67.0 [60.0–73.0]	66.5 [59.0–75.0]	1.00 [0.98–1.02]	0.749
Male sex	482 (54.6%)	57 (52.8%)	0.93 [0.62–1.39]	0.8
eGFR, mL/min/1.73 m^2^	76.0 [63.0–90.0]	75.0 [61.0–89.0]	1.00 [0.99–1.01]	0.534
BMI, kg/m^2^	27.9 [25.0–31.1]	28.7 [25.1–32.0]	1.02 [0.98–1.06]	0.248
CRP, mg/L	3.60 [2.20–6.60]	3.95 [2.64–6.60]	0.99 [0.98–1.01]	0.596
Fibrinogen, g/L	3.22 [2.80–3.93]	3.34 [3.00–3.99]	1.18 [0.97–1.43]	0.121
Total cholesterol, mmol/L	4.88 [4.03–5.77]	4.88 [4.00–5.51]	0.93 [0.79–1.09]	0.489
LDL-C, mmol/L	3.09 [2.30–3.84]	2.98 [2.42–3.61]	0.87 [0.72–1.05]	0.243
Triglycerides, mmol/L	1.30 [1.09–1.81]	1.30 [1.04–1.57]	0.91 [0.74–1.11]	0.384
LVEF, %	59.0 [51.0–64.0]	59.0 [48.8–65.0]	1.00 [0.98–1.02]	0.923
Type 2 diabetes	218 (24.7%)	29 (26.9%)	1.12 [0.70–1.75]	0.618
Obstructive CAD	459 (52.0%)	65 (60.2%)	1.39 [0.93–2.11]	0.108
Prior MI	263 (29.8%)	36 (33.3%)	1.18 [0.76–1.80]	0.448
Smoking	153 (17.3%)	18 (16.7%)	0.96 [0.55–1.61]	0.883
Hemoglobin, g/L	140 [129–151]	139 [126–151]	1.00 [0.99–1.01]	0.569
Mortality	130 (14.7%)	14 (13.0%)	0.87 [0.46–1.53]	0.73
PRU	150 [120–188]	230 [217–255]	-	<0.001
DAPT	540 (61.2%)	50 (46.3%)	0.55 [0.37–0.82]	0.004
Ticagrelor	230 (26.0%)	6 (5.6%)	0.17 [0.07–0.36]	<0.001
Clopidogrel	310 (35.1%)	44 (40.7%)	1.27 [0.84–1.91]	0.299

All abbreviations as in Table A3.

**Table A4:** Full descriptive statistics across PRU quartiles (monotherapy subgroup; *n* = 402).

Variable	Q1 (*n* = 200)	Q2 (*n* = 38)	Q3 (*n* = 91)	Q4 (*n* = 73)	*p*-Overall
Median PRU	120 [110–120]	155 [150–155]	190 [180–200]	220 [210–235]	<0.001
Age, years	66.0 [60.0–72.0]	67.5 [57.0–73.8]	68.0 [61.0–73.5]	66.0 [60.0–75.0]	0.707
Male sex	89 (44.5%)	17 (44.7%)	37 (40.7%)	37 (50.7%)	0.646
eGFR, mL/min/1.73 m^2^	77.0 [65.0–91.0]	82.0 [73.2–96.0]	74.0 [61.5–90.0]	78.0 [61.0–90.0]	0.327
BMI, kg/m^2^	28.0 [25.0–31.2]	27.8 [25.4–31.1]	28.4 [25.4–30.8]	29.7 [25.4–32.7]	0.381
CRP, mg/L	3.30 [2.80–4.60]	3.30 [2.59–4.78]	3.30 [2.30–4.50]	3.30 [2.25–5.00]	0.914
Fibrinogen, g/L	3.39 [3.00–4.00]	3.21 [2.81–3.68]	3.20 [2.79–3.84]	3.31 [2.94–4.00]	0.19
Total cholesterol, mmol/L	4.92 [4.30–5.70]	4.96 [3.79–5.70]	4.75 [3.85–5.73]	4.97 [4.16–5.70]	0.819
LDL-C, mmol/L	3.00 [2.30–3.72]	3.10 [2.43–3.69]	2.96 [2.35–3.61]	2.85 [2.40–3.50]	0.788
Triglycerides, mmol/L	1.30 [1.10–1.42]	1.30 [1.13–1.48]	1.30 [1.12–1.50]	1.30 [1.04–1.40]	0.481
LVEF, %	60.0 [53.0–64.0]	58.0 [50.0–64.8]	60.0 [51.2–66.0]	59.0 [51.0–65.0]	0.916
Hemoglobin, g/L	141 [130–151]	138 [129–150]	138 [126–148]	141 [129–148]	0.397
Type 2 diabetes	34 (17.0%)	10 (26.3%)	23 (25.3%)	18 (24.7%)	0.247
Obstructive CAD	63 (31.5%)	13 (34.2%)	27 (29.7%)	34 (46.6%)	0.091
Prior MI	46 (23.0%)	6 (15.8%)	18 (19.8%)	19 (26.0%)	0.592
Smoking	30 (15.0%)	5 (13.2%)	7 (7.7%)	12 (16.4%)	0.312
COPD	5 (2.5%)	1 (2.6%)	0 (0.0%)	5 (6.9%)	0.052
COVID-19 history	39 (19.5%)	6 (15.8%)	17 (18.7%)	12 (16.4%)	0.913
D-dimer	190 [100–358]	180 [100–330]	188 [100–315]	200 [120–350]	0.871
Troponin I	0.001 [0.001–0.002]	0.002 [0.001–0.003]	0.001 [0.001–0.003]	0.001 [0.001–0.003]	0.556
HRPR (PRU ≥ 208)	0 (0.0%)	0 (0.0%)	0 (0.0%)	58 (79.5%)	<0.001
Mortality	32 (16.0%)	3 (7.9%)	13 (14.3%)	8 (11.0%)	0.491
Statin + ezetimibe	14 (7.0%)	2 (5.3%)	9 (9.9%)	2 (2.7%)	0.335

All patients in this subgroup received antiplatelet monotherapy. Q1 = lowest PRU quartile; Q4 = highest PRU quartile. All abbreviations as in Table A3.

**Table A5:** Differences between survivors and non-survivors in the PRU ≥ 155 subgroup.

Variable	Survived (*N* = 480)	Died (*N* = 66)	OR	*p* (Univariate)	*p* (Overall)	*N*
Age	65.0 [59.0–72.0]	75.0 [71.0–80.8]	1.14 [1.10–1.18]	<0.001	<0.001	546
Sex				0.016		546
Female	204 (42.5%)	39 (59.1%)	Reference	Reference		
Male	276 (57.5%)	27 (40.9%)	0.51 [0.30–0.86]	0.012		
eGFR	77.0 [65.0–92.0]	65.5 [53.0–85.5]	0.97 [0.96–0.99]	<0.001	<0.001	544
BMI	28.0 [25.0–31.2]	26.5 [25.0–29.3]	0.93 [0.88–0.99]	0.017	0.008	546
CRP	3.60 [2.20–6.54]	5.12 [2.20–10.3]	1.01 [1.00–1.01]	0.051	0.092	543
Total cholesterol	4.90 [4.10–5.80]	4.70 [3.79–5.45]	0.79 [0.64–0.98]	0.032	0.034	546
LDL-C	3.10 [2.42–3.90]	2.92 [2.32–3.68]	0.82 [0.64–1.05]	0.117	0.097	545
Triglycerides	1.30 [1.11–2.05]	1.15 [0.85–1.39]	0.38 [0.23–0.64]	<0.001	<0.001	546
Fibrinogen	3.16 [2.75–3.84]	3.44 [2.96–4.16]	1.26 [1.00–1.60]	0.052	0.029	545
Type 2 diabetes				0.891		546
No	357 (74.4%)	48 (72.7%)	Reference	Reference		
Yes	123 (25.6%)	18 (27.3%)	1.09 [0.60–1.92]	0.764		
Prediabetes				1.000		546
No	465 (96.9%)	64 (97.0%)	Reference	Reference		
Yes	15 (3.12%)	2 (3.03%)	1.03 [0.15–3.81]	0.969		
COVID-19 history				0.808		546
No	375 (78.1%)	53 (80.3%)	Reference	Reference		
Yes	105 (21.9%)	13 (19.7%)	0.88 [0.44–1.64]	0.705		
COPD				0.750		546
No	460 (95.8%)	63 (95.5%)	Reference	Reference		
Yes	20 (4.17%)	3 (4.55%)	1.14 [0.25–3.48]	0.840		
Coronary artery disease severity				0.090		546
Non-obstructive	232 (48.3%)	24 (36.4%)	Reference	Reference		
Obstructive	248 (51.7%)	42 (63.6%)	1.63 [0.96–2.82]	0.069		
D-dimer	220 [132–505]	245 [150–668]	1.00 [1.00–1.00]	0.049	0.381	545
Troponin	0.003 [0.001–0.013]	0.005 [0.002–0.052]	1.00 [0.98–1.02]	0.756	0.034	545
LVEF (%)	59.0 [51.8–64.0]	53.5 [42.2–61.0]	0.96 [0.94–0.98]	0.001	0.001	546
Smoking				0.008		546
No	382 (79.6%)	62 (93.9%)	Reference	Reference		
Yes	98 (20.4%)	4 (6.06%)	0.26 [0.08–0.65]	0.002		
Hemoglobin	140 [129–151]	134 [122–145]	0.98 [0.96–0.99]	0.001	0.003	546
Prior MI				0.043		546
No	346 (72.1%)	39 (59.1%)	Reference	Reference		
Yes	134 (27.9%)	27 (40.9%)	1.79 [1.04–3.03]	0.035		
PRU	200 [175–205]	194 [176–204]	1.00 [0.99–1.01]	0.902	0.984	546
Statin therapy				1.000		546
Statin	444 (92.5%)	61 (92.4%)	Reference	Reference		
Statin + ezetimibe	36 (7.50%)	5 (7.58%)	1.04 [0.34–2.54]	0.943		
HRPR				0.883		546
Therapeutic window	386 (80.4%)	52 (78.8%)	Reference	Reference		
BORT outside range	94 (19.6%)	14 (21.2%)	1.11 [0.57–2.05]	0.742		
Antiplatelet therapy:				1.000		546
Monotherapy	166 (34.6%)	23 (34.8%)	Reference	Reference		
Dual therapy	314 (65.4%)	43 (65.2%)	0.99 [0.58–1.72]	0.958		
Ticagrelor use				0.113		546
No	369 (76.9%)	57 (86.4%)	Reference	Reference		
Yes	111 (23.1%)	9 (13.6%)	0.53 [0.24–1.06]	0.075		
Clopidogrel use				0.204		545
No	276 (57.6%)	32 (48.5%)	Reference	Reference		
Yes	203 (42.4%)	34 (51.5%)	1.44 [0.86–2.43]	0.165		

Non-linear relationship between platelet reactivity and all-cause mortality in coronary artery disease: evidence for an optimal therapeutic window. Abbrevations: GFR = glomerular filtration rate, CRP = C-reactive protein, BMI = body mass index, LDL-C = low-density lipoprotein cholesterol, COPD = Chronic obstructive pulmonary disease, LVEF = left ventricular ejection fraction, MI = myocardial infarction, PRU = P2Y12 reaction units, HRPR = high residual platelet reactivity.
